# Supplementary material for: The Usefulness of a Massive Open Online Course about Postural and Technological Adaptations to Enhance Academic Performance and Empathy in Health Sciences Undergraduates
Source: Int J Environ Res Public Health. 2021 Oct 12;18(20):10672. doi: 10.3390/ijerph182010672 (PMC8536090; doi:10.3390/ijerph182010672)
Supplement: Supplementary file 1 [file ijerph-18-10672-s001.zip › ijerph-1387545-supplementary.pdf]

Table S1: Description of Assessing a Massive Online Course Rubric.

Category	Needs improvement (0)	Basic (1)	Good (2)	Excellent (3)	SCORE
1. Knowledge about disability					
2. Identifying the differences between different pathological posture schemes					
3. To know what a support product is					
4. Make a postural adaptation					
5. Concept of Virtual Reality					
6. Philosophy of low-cost					
7.Technological devices of low-cost postural intervention					
8. The importance of adapted games					
9. Assessment critical thinking					
10. Organization					
11.Professor provides Feedback/ Encouragement on Discussion Boards					
**TOTAL SCORE**					
